# Breastfeeding Self-Efficacy, Personal Well-Being and Related Factors in Pregnant Women Living in a District of Istanbul

**DOI:** 10.3390/nu15214541

**Published:** 2023-10-26

**Authors:** Mehmet Sait Değer, Mehmet Akif Sezerol, Zeynep Meva Altaş

**Affiliations:** 1 Department of Public Health, School of Medicine, Hitit University, Çorum 19040, Türkiye; mehmetsaitdeger@hitit.edu.tr; 2 Epidemiology Program, Institute of Health Sciences, Istanbul Medipol University, Istanbul 34820, Türkiye; masezerol@gmail.com; 3 Department of Public Health, School of Medicine, Istanbul Medipol University, Istanbul 34820, Türkiye; 4 Sultanbeyli District Health Directorate, Istanbul 34935, Türkiye; 5 Ümraniye District Health Directorate, Istanbul 34764, Türkiye

**Keywords:** breastfeeding, self-efficacy, well-being, pregnant women, Prenatal Breastfeeding Self-Efficacy Scale, Personal Well-Being Scale

## Abstract

In this study, we examined breastfeeding self-efficacy levels, well-being and sociodemographic factors in pregnant women. The population of this descriptive study consisted of women with a pregnancy of 27 weeks or more in the Sultanbeyli district of Istanbul, Türkiye. A questionnaire was administered via telephone calls to pregnant women aged 18 years and older. The first part of the questionnaire included questions regarding breastfeeding history and sociodemographic information. The second part included the Prenatal Breastfeeding Self-Efficacy Scale, and the last part included the Personal Well-Being Scale. Higher scale scores indicate higher levels of self-efficacy and well-being. In total, 385 women participated in the study. The median age of the pregnant women was 28.0 years (18.0–43.0). The median gestational week was 33.0 (27.0–42.0). Among women who had received breastfeeding counseling, those with a higher level of knowledge about breastfeeding had higher breastfeeding self-efficacy (*p* < 0.05). Women with better economic status also had higher well-being scores (*p* < 0.05). There was a positive correlation between well-being and breastfeeding self-efficacy approaching the statistical significance level (*p* = 0.052). It is important to consider factors that may be associated with women’s well-being and self-efficacy.

## 1. Introduction

Breastfeeding is one of the most effective ways to ensure a child’s well-being and survival. Breast milk is a safe and clean food and contains antibodies that help protect against various childhood diseases [1]. It contains many complex proteins, lipids and carbohydrates to meet the needs of the baby [2]. Essential nutrients such as vitamins are transferred from the mother’s blood circulation to the milk via the mammary gland, ultimately reaching the baby through breastfeeding. Adequate vitamin and mineral contents in breast milk are very important for infant development [3]. In addition to being a source of nutrition for infants, breast milk also contains numerous biologically active components. These molecules play various roles in guiding the development of both the infant’s immune system and gut microbiota [2].

The U.S. Dietary Guidelines for Americans recommend that infants should be exclusively breastfed for approximately the first six months and then breastfed with appropriate complementary foods until the child is twelve months of age or older [4]. The American Academy of Pediatrics and the World Health Organization (WHO) also recommend exclusive breastfeeding for the first six months, followed by appropriate complementary foods and continued breastfeeding until two years of age or older [1]. The WHO encourages breastfeeding as the best source of nutrition for infants and young children. The WHO is working toward increasing the rate of exclusive breastfeeding for the first six months to at least 50% by the year 2025 [1]. Babies should be breastfed on demand. In other words, the mother should breastfeed her baby as often as the child wants during the day and night. Babies should not use baby bottles or pacifiers. From six months of age, children should start eating safe and adequate complementary foods, and breastfeeding should be continued at the age of two and beyond [1,5].

Contrary to recommendations, less than half of infants under six months of age are exclusively breastfed [1]. According to data from the 2018 Turkish Demographic and Health Survey, 98% of children born in Türkiye were breastfed. This rate was 96.4% in 2013. According to the 2018 Turkish Demographic and Health Survey data, the percentage of infants who were exclusively breastfed for the first six months was 41%. The median duration of breastfeeding was 16.7 months, and the median duration of exclusive breastfeeding was 1.8 months [6].

The benefits of breast milk are multifaceted. Breastfeeding provides many benefits to both the mother and the baby. In general, breast milk is extremely important in terms of improving and maintaining health and well-being, providing adequate and balanced nutrition. Moreover, breast milk contributes to the healthy growth and development of the baby and has positive effects on the immune system [7,8,9,10,11]. The risks of many diseases including asthma, obesity, diabetes, celiac disease, severe lower respiratory tract disease, atopic dermatitis, Crohn’s disease and ulcerative colitis are lower in breastfed babies [4,12,13]. Breastfeeding mothers have been reported to have lower risks of breast cancer, endometrial cancer, ovarian cancer, thyroid cancer, diabetes and hypertension [4,12]. In addition, breastfeeding facilitates a mother’s return to her pre-pregnancy weight [14]. A mother’s sense of bonding with her baby is also strengthened by breastfeeding [7]. In terms of women’s and children’s health, there is a need to continue and support breastfeeding and to disseminate programs that support breastfeeding with breast milk [15].

Breastfeeding provides numerous positive outcomes not only for the health of the mother and child but also for the general community. Some of these benefits include improving and preserving community health, reducing healthcare expenditures, decreasing the demand for workforce resources dedicated to child illnesses and treatments, and reducing absenteeism, the need for leave, and financial losses. These advantages highlight the multifaceted benefits of breastfeeding, not only for individual health but also for broader society [16].

Despite all the known benefits of breast milk and breastfeeding, unfortunately, women around the world continue to face significant barriers to breastfeeding. Understanding these barriers is crucial to increasing the proportion of breastfed infants. Common barriers to breastfeeding include a lack of information, social norms, a lack of family support, embarrassment, problems related to the act of breastfeeding (such as pain and mastitis), problems related to work and childcare, and barriers related to health services [17]. Understanding these barriers is extremely important in identifying the priorities for interventions aimed at promoting breast milk.

The desire of mothers to breastfeed and their self-efficacy in this regard are extremely important for maintaining breastfeeding [18]. Self-efficacy is defined as “people’s judgments about their ability to plan and perform actions that can allow them to achieve a certain performance” [19]. Self-efficacy plays a pivotal role in influencing various behaviors, including the ability to breastfeed effectively [20]. Breastfeeding self-efficacy encompasses a mother’s level of confidence in her breastfeeding capabilities, and it exerts a significant influence on both the initiation and duration of breastfeeding, making it a factor that can be modified or improved [21,22]. Self-efficacy expectations for breastfeeding include the mother’s confidence in her ability to perform behaviors that can enable her to successfully breastfeed her baby [23]. There are many factors that affect breastfeeding self-efficacy, one of which is the mother’s well-being. In addition, sociodemographic factors may affect both breastfeeding self-efficacy and the well-being of the mother [24]. In this study, we aimed to examine breastfeeding self-efficacy levels and the well-being of women in the last stage of pregnancy and to evaluate sociodemographic aspects that may be related to these factors.

## 2. Materials and Methods

### 2.1. Study Type and Design

The population of this descriptive study consisted of women with a pregnancy of 27 weeks or more who were registered with a family physician in the Sultanbeyli district of Istanbul. The Sultanbeyli district has the lowest socioeconomic development index compared with the other districts of Istanbul [25]. The study aimed to include pregnant women aged 18 years and older who had a registered phone number in the system and were not foreign nationals. The number of all pregnant women registered in the district during the study period was 1043. Each of these women was called twice for the study. The study used a questionnaire administered via telephone calls with pregnant women who answered the calls and agreed to participate in our study.

### 2.2. Questionnaire

The first part of the questionnaire included questions related to sociodemographic characteristics and breastfeeding history. The second part included the Prenatal Breast-feeding Self-Efficacy Scale, and the last part included the Personal Well-Being Scale.

#### 2.2.1. Prenatal Breastfeeding Self-Efficacy Scale (PBSES)

The Prenatal Breastfeeding Self-Efficacy Scale (PBSES) was developed in 2006 by Wells et al. to determine the breastfeeding self-efficacy perceptions of pregnant women [26]. Aydin and Pasinlioglu adapted the scale into Turkish in 2016 [27]. The scale consists of 20 items and subitems in total. It has no subscales and is a 5-point Likert type scale. The items on the scale are “Not all sure” (1 point), “Slightly sure” (2 points), “Fairly sure” (3 points), “Very sure” (4 points), and “Completely sure” (5 points). There are no reverse-scored items in the scale. The minimum obtainable score from the scale is 20, and the highest score is 100. A cut-off point on the scale is not available. Higher scores obtained from the scale indicate higher perceptions of breastfeeding self-efficacy among women.

#### 2.2.2. Personal Wellbeing Index—Adult (PWI-A)

The Personal Wellbeing Index—Adult (PWI-A) was developed by the International Wellbeing Group [28]. It is a thematic and 11-point Likert-type (scores range between 0 and10 points) measurement tool that aims to measure well-being through individuals’ satisfaction levels in eight life domains. The life domains measured by the PWI-A form are the standard of living, health, achieving in life, relationships, safety, community connectedness, future security, and religion/spirituality. Each of the eight life domains that the PWI-A form aims to measure is measured with a total of eight questions, using a single question for each life domain. The lowest score that can be obtained from the 11-point Likert-type scale (0 (Not at all satisfied)—5 (Undecided)—10 (Completely satisfied)) is 0, and the highest score is 80. Meral examined the psychometric properties of the PWI-A form on an adult sample in Türkiye in 2014 [29].

### 2.3. Statistical Analysis

This study employed SPSS (Statistical Package for Social Sciences) for Windows 25.0 to carry out a statistical analysis and data recording. Median, minimum and maximum values, numbers (*n*) and percentages (%) were used for the presentation of descriptive data. The conformity of continuous variables to a normal distribution was examined using visual (histogram and probability charts) and analytical methods (the Kolmogorov-Smirnov and Shapiro-Wilk tests). Student’s *t* test was used to compare the two groups for normally distributed data, and an ANOVA was used to compare more than two groups with normal distributions. Pearson’s chi-squared test was used for the statistical analysis of categorical data. The statistical significance level was determined to be *p* < 0.05.

### 2.4. Ethics

To conduct the study, ethics committee approval was obtained from the Istanbul Medipol University Non-Interventional Clinical Research Ethics Committee, decision number 647, on 10 August 2023. Prior to the study, verbal informed consent was obtained from the participants during telephone calls. After receiving their consent, the questionnaire was administered.

## 3. Results

**Figure 1:**
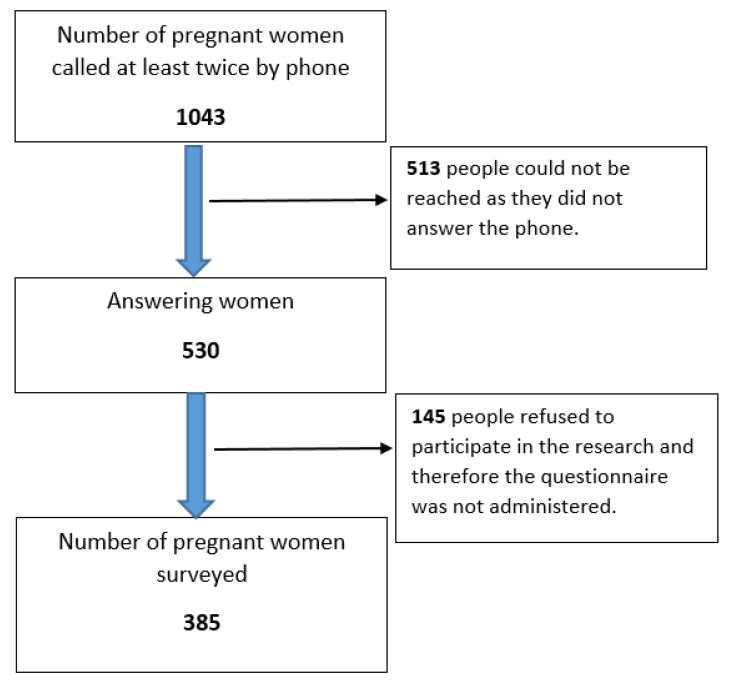
Participating pregnant women.

In our study, there were 1043 pregnant women registered in the district during the study period. Each of these women was called at least twice via telephone. Of the women called, 513 could not be reached as they did not answer the phone. Of the women, 530 answered, and 385 provided verbal consent to participate in the study and answered the questionnaire during the phone call (Figure 1).

A questionnaire was administered to 385 pregnant women. The median age was 28.0 years (18.0–43.0). The median gestational week was 33.0 (27.0–42.0). Of the participants, 11.4% (*n* = 44) had chronic diseases, 33.8% (*n* = 130) were experiencing their first pregnancy and 47.0% (*n* = 181) received breastfeeding counseling. Other sociodemographic characteristics of the pregnant women are shown in Table 1.

The pregnant women were posed questions about breastfeeding and whether they knew the related information. All pregnant women stated that they knew that infants should be given only breast milk for the first six months. The percentage of pregnant women who knew that breastfeeding should be continued until the age of two years with complementary food was 99.5% (*n* = 383). Of the women, 98.7 (*n* = 380) knew that breastfeeding creates a positive emotional bond between mother and baby. The percentage of those who knew that breastfeeding protects the mother from uterine and breast cancer was 84.4% (*n* = 325). The percentage of pregnant women who correctly answered all the questions about breastfeeding was 70.9% (*n* = 273). Table 2 shows the responses of the pregnant women to other similar questions about breastfeeding.

Eight subdimensions of the PWI-A Index were administered to the pregnant women. When the median values of the scores obtained for each subdimension were analyzed, the highest scores were obtained in the dimensions of relationships, safety, and religion/spirituality, while the lowest score median was observed in the standard of living health dimension (Table 3).

The median total score of the PWI-A was 67.0 (5.0–80.0). The median PBSES score was 96.0 (58.0–100.0). Factors associated with the PBSES and PWI-A scores were evaluated. When evaluating economic status, those whose income was equal to their expenses and those whose income was higher than their expenses were considered to have high economic status. Pregnant women whose income was less than their expenses were considered to have low economic status. The PWI-A scores of pregnant women with high economic status were significantly higher (*p* < 0.001). Pregnant women who received breastfeeding counseling had significantly higher PBSES scores (*p* = 0.042). The PBSES scores of pregnant women who answered all the questions about breastfeeding correctly were also significantly higher (*p* < 0.001). The presence of chronic disease, education level, and first pregnancy had no significant effects on either PBSES or PWI-A scores (*p* > 0.05). Table 4 shows factors associated with PBSES and PWI-A scores.

Correlations between age, gestational week, the number of people at home, the number of children and the PWI-A and PBSES scores were also evaluated. There was no significant correlation between age, gestational week, the number of people living at home and the number of children and the PBSES and PWI-A scores (*p* > 0.05). The positive correlation between the PBSES and PWI-A scores approached statistical significance (r = 0.099, *p* = 0.052) (Table 5).

## 4. Discussion

Pregnant women represent a vulnerable group that should be prioritized in public health interventions [30]. It is important to identify the maternal and infant health requirements that women need in preparation for childbirth. Breastfeeding is an extremely important and vital action in terms of maternal and infant health and social impact. For this reason, it is necessary to maintain breastfeeding with recommendations and to determine the factors affecting breastfeeding. Breastfeeding self-efficacy is one of the factors that may affect mothers’ breastfeeding success. In this study, we aimed to assess breastfeeding self-efficacy and related sociodemographic factors among pregnant women in their last trimester and to evaluate the relationship between well-being and breastfeeding self-efficacy.

Studies have shown that mothers with high levels of breastfeeding self-efficacy continue breastfeeding longer, and exclusive breastfeeding rates are higher in women with high levels of breastfeeding self-efficacy [23,31]. In a study conducted among postpartum women in Türkiye, a positive correlation was found between breastfeeding self-efficacy and positive breastfeeding attitudes [22]. The same study suggested that as breastfeeding self-efficacy increased, women’s postpartum depression scores decreased. The results of these studies reflect the importance of breastfeeding self-efficacy. Breastfeeding self-efficacy can be affected by some sociodemographic factors. In a study conducted with pregnant women, breastfeeding self-efficacy was associated with women’s educational level, occupational status and previous breastfeeding experience [32]. In our study, we aimed to investigate the factors related to breastfeeding self-efficacy and found that pregnant women who received breastfeeding counseling and had better knowledge about breastfeeding had significantly higher PBSES scores. In a randomized controlled study in the literature, mothers who received breastfeeding counseling had higher self-efficacy scores than those who did not, similar to our study [33]. Similarly, a meta-analysis study showed that theoretical breastfeeding education provided to pregnant women in a health center contributed positively to breastfeeding self-efficacy [34]. In addition to self-efficacy, breastfeeding counseling was reported to have a positive effect on breastfeeding behavior itself in one of the studies [35]. Providing breastfeeding counseling can be expected to have a positive effect on breastfeeding behavior, because it is a skill that requires acquisition [36] and breastfeeding counseling involves the hands-on practice of breastfeeding skills, assisting mothers in holding their infants correctly, managing common breast issues, such as nipple injuries, engorgement and mastitis [37]. Thus, our study, along with studies in the literature, highlights the importance of breastfeeding counseling [38]. However, in our study, only 47.0% of the women received breastfeeding counseling. In this regard, there is a need to promote the dissemination of breastfeeding counseling services and increase access for pregnant women.

In our study, pregnant women were asked questions about breastfeeding and whether they knew the answers. The percentage of pregnant women who correctly answered all questions about breastfeeding was 70.9%. The PBSES scores of the pregnant women who answered all the questions about breastfeeding correctly were also significantly higher. In a similar study in the literature, mothers with a higher level of breastfeeding knowledge were reported to have greater breastfeeding self-efficacy [39]. In addition, positive thoughts and attitudes about breastfeeding during pregnancy contribute to the development of confidence in breastfeeding and make it easier to overcome difficulties [40]. Similarly, in a different study, possessing sufficient knowledge about breastfeeding and having positive attitudes toward breastfeeding were positively correlated with increased levels of breastfeeding self-efficacy [41]. In our study, approximately 30.0% of the mothers had insufficient knowledge about breastfeeding, indicating the need for informative sessions about breastfeeding as part of prenatal care programs. This can promote women’s perceptions of breastfeeding self-efficacy.

The well-being of the mother is extremely important for successful breastfeeding. In one study, the stressful life events experienced during pregnancy increased the risk of the early cessation of breastfeeding [42]. In our study, the PWI-A scores of pregnant women with high economic status were significantly higher. In a large sample study in the literature which was conducted in adults, similar to our results, the PWI scores of those with lower socioeconomic status were lower [43]. In addition, the positive correlation between the PBSES and PWI-A scores approached statistical significance in our study. Self-efficacy was reported to be a contributing factor to well-being in studies conducted in different populations [44,45]. Therefore, high levels of breastfeeding self-efficacy may contribute to well-being in pregnant women in a similar way. To our knowledge, there is no study in the literature investigating well-being and breastfeeding self-efficacy in pregnant women. Therefore, our study sheds light on this issue in the literature. There is a need for studies with larger sample sizes to examine the relationship between the well-being of pregnant women and breastfeeding self-efficacy. When planning interventions and policies related to breastfeeding, it is important to consider factors that may be associated with women’s well-being and self-efficacy and to adopt multidisciplinary approaches. According to the results of our study, it is noteworthy that addressing the economic needs of pregnant women and postpartum mothers, as well as addressing gaps in their knowledge about breastfeeding, is essential in interventions related to breastfeeding.

### Limitations and Strengths

We conducted our study with pregnant women in the last trimester from a single district in Istanbul. This situation reduces the generalizability of our study findings. Additionally, the low response rate among the pregnant women contacted and those who agreed to participate via telephone may limit the representativeness of the pregnant women in the district. Information about the breastfeeding behaviors of nonparticipating pregnant women and their perceptions of self-efficacy may differ from those who participated in the study. A postpartum evaluation would have been useful given that a large number of the women interviewed were experiencing their first pregnancy. Despite these limitations, our study provides a wide range of data, including well-being and self-efficacy concepts associated with breastfeeding behavior, as well as sociodemographic characteristics, which are highly relevant to maternal and child health. This strengthens the study’s overall significance.

## 5. Conclusions

In our study, among women receiving breastfeeding counseling, those with a higher level of knowledge about breastfeeding had a higher level of breastfeeding self-efficacy. Women with a better economic status also had higher well-being scores. Furthermore, there was a positive correlation between well-being and breastfeeding self-efficacy near the statistical significance level. Larger prospective, qualitative studies are necessary to further explore the relationship between maternal well-being and breastfeeding self-efficacy. Based on the findings of our research, it is crucial to emphasize that any efforts and interventions aimed at promoting breastfeeding should not overlook the significance of addressing both the economic requirements of pregnant women and postpartum mothers, as well as bridging knowledge gaps concerning breastfeeding. These aspects should be considered integral components of any comprehensive strategy aimed at promoting and supporting breastfeeding practices The promotion of breastfeeding through various interventions, educational programs, and services such as breastfeeding counseling can lead to substantial enhancements in the nutritional, immune, psychosocial, and economic well-being of both children and women, thereby contributing to improved public health outcomes.

## Figures and Tables

**Table 1:** Sociodemographic characteristics and pregnancy features of the participants.

	Median (Min–Max)
Age (years)	28.0 (18.0–43.0)
Gestational week	33.0 (27.0–42.0)
Number of people living at home	3.0 (2.0–10.0)
Number of children	1.0 (0–4.0)
	N (%)
Presence of chronic disease	44 (11.4)
Smoking	28 (7.3)
Alcohol use	1 (0.3)
Economical level *	N (%)
Income less than expenditure	180 (46.8)
Income more than expenditure	14 (3.6)
Equal income and expenditure	127 (33.0)
Educational level	N (%)
Illiterate	7 (1.8)
Only literate	13 (3.4)
Primary school	126 (32.7)
High school	115 (29.9)
University/Master	124 (32.2)
First pregnancy	130 (33.8)
Receiving breastfeeding counseling	181 (47.0)

* Out of women, 64 did not answer the question about their economic level.

**Table 2:** Questions about breastfeeding knowledge.

		N (%) *
Do you know that children should be exclusively breastfed for the first six months after birth?	Yes	385 (100)
After six months, do you know that your child should be breastfed with supplementary foods until at least two years of age?	Yes	383 (99.5)
Do you know that breastfeeding creates a positive emotional bond between mother and baby?	Yes	380 (98.7)
Do you know that breastfeeding protects the mother against uterine and breast cancer?	Yes	325 (84.4)
Do you know that breastfeeding mothers return to their old weight more quickly?	Yes	345 (89.6)
Do you know that breastfeeding hormones relax the mother and improve sleep quality?	Yes	325 (84.4)
Do you know that breastfeeding whenever the baby wants increases the amount of milk?	Yes	358 (93.0)
Do you know that you need to breastfeed your baby often, drink plenty of fluids and get enough rest to ensure adequate milk supply?	Yes	380 (98.7)
Do you know that when breastfeeding your baby, you should give the whole brown part of your breast, not the tip?	Yes	370 (96.1)
The skin-to-skin contact between mother and baby in the first hour after birth helps the milk to come down early and strengthens the bond between baby and mother. Do you know this?	Yes	365 (94.8)
Do you know that keeping mother and baby together for 24 h immediately after birth increases the sense of trust between baby and mother?	Yes	365 (94.8)
Knowing all the questions about breastfeeding	Yes	273 (70.9)

* These are the percentages of participants who answered “yes”.

**Table 3:** The median scores of the PWI-A subdimensions.

	Median (Min–Max)
Standard of living health	6.0 (0–10.0)
Health	9.0 (0–10.0)
Achieving in life	9.0 (0–10.0)
Relationships	10.0 (0–10.0)
Safety	10.0 (0–10.0)
Community connectedness	9.0 (0–10.0)
Future security	8.0 (0–10.0)
Religion/spirituality	10.0 (0–10.0)

**Table 4:** Factors associated with PBSES and PWI-A scores.

		PBSES		PWI-A	
		Median (Min–Max)	*p* Value	Media (Min–Max)	*p* Value
Presence of chronic disease	No	96.0 (58.0–100)	0.878	68.0 (20.0–80.0)	0.132
	Yes	94.0 (70.0–100)		65.0 (5.0–80.0)	
Education level	Below high school	94.0 (58.0–100)	0.055	67.5 (5.0–80.0)	0.546
	High school and above	96.0 (62.0–100)		67.0 (20.0–80.0)	
Economical level	Low	95.5 (58.0–100)	0.598	63.0 (5.0–80.0)	<0.001
	High	96.0 (66.0–100)		70.0 (34.0–80.0)	
Receiving breastfeeding counseling	No	94. 0 (61.0–100)	0.042	68.0 (5.0–80.0)	0.273
	Yes	96.0 (58.0–100)		67.0 (20.0–80.0)	
First pregnancy	No	95.0 (58.0–100)	0.113	66.0 (5.0–80.0)	0.052
	Yes	96.0 (70.0–100)		69.0 (20.0–80.0)	
Answering all the questions about breastfeeding	No	93.0 (62.0–100)	<0.001	66.0 (22.0–80.0)	0.814
	Yes	96.0 (58.0–100)		68.0 (5.0–80.0)	

PBSES: Prenatal Breastfeeding Self-Efficacy Scale, PWI-A: Personal Wellbeing Index—Adult.

**Table 5:** Correlations between age, gestational week, the number of people at home, the number of children and the PWI-A and PBSES scores.

		PBSES	PWI-A
Age	Correlation coefficient (r)	−0.022	−0.096
	*p* value	0.675	0.060
	N	380	380
Gestational week	Correlation coefficient (r)	−0.055	0.044
	*p* value	0.284	0.397
	N	379	379
Number of people living at home	Correlation coefficient (r)	−0.078	−0.090
	*p* value	0.128	0.078
	N	385	385
Number of children	Correlation coefficient (r)	−0.058	−0.077
	*p* value	0.253	0.132
	N	385	385
PBSES	Correlation coefficient (r)	1.000	0.099
	*p* value		0.052
	N	385	385
PWI-A	Correlation coefficient (r)	0.099	1.000
	*p* value	0.052	
	N	385	385

PBSES: Prenatal Breastfeeding Self-Efficacy Scale, PWI-A: Personal Wellbeing Index—Adult.

## Data Availability

Not applicable.
